# Spontaneous extradural hematoma in a Sickle cell Beta Thalassemia patient—A rare complication

**DOI:** 10.1002/ccr3.6917

**Published:** 2023-02-03

**Authors:** Prasanna Ghimire, Pragya Gautam Ghimire

**Affiliations:** ^1^ Department of Radiology Nepalgunj Medical College and Teaching Hospital Kohalpur Nepal; ^2^ Department of Pathology Nepalgunj Medical College and Teaching Hospital Kohalpur Nepal

**Keywords:** extradural, hematoma, sickle cell disease, thalassemia

## Abstract

Spontaneous extradural hematoma in Sickle cell disease is rare neurological complication with few cases reported in the English literature. We report a case of a 16‐year‐old male patient who was previously diagnosed with Sickle Cell Beta Thalassemia and presented with severe headache and vomiting for 3 days. An emergency CT scan of the head demonstrated right‐sided acute parietal extradural hematoma with mass effect. Patient underwent emergent craniotomy with evacuation of the hematoma. Patient recovered completely. Although calvarial infarction has been associated with extradural hematoma, an absence of it makes our case distinct. A high index of suspicion should be made in SCD patients for possibility of EDH in progressive headache.

## Abstract 2

Although neurological complications are rare in SCD patients in the Asian population, progressive headache should be investigated for associated neurosurgical emergencies in the targeted population.

## 1 INTRODUCTION

Sickle cell disease (SCD) is an autosomal recessive hemoglobinopathy due to monogenetic disorder. The inheritance can be in the homozygous state (Hb SS) or compound heterozygous state where an association with another globin gene mutation is noted. Global variation in the cases of SCD is noted with highest frequency noted in the sub‐Saharan Africa, central India, and the Middle East.^1^ SCD often presents as multiple organ disorder with central nervous system complication considered most dreaded.^2^ Although chronic headache, epilepsy, ischemic or hemorrhagic stroke, cognitive impairment due to chronic anemia, Moyamoya disease, hypoxia, and silent infarcts are seen in SCD, spontaneous extradural hematoma has rarely been reported in the English literature.^3^

## 2 CASE PRESENTATION

**FIGURE 1:**
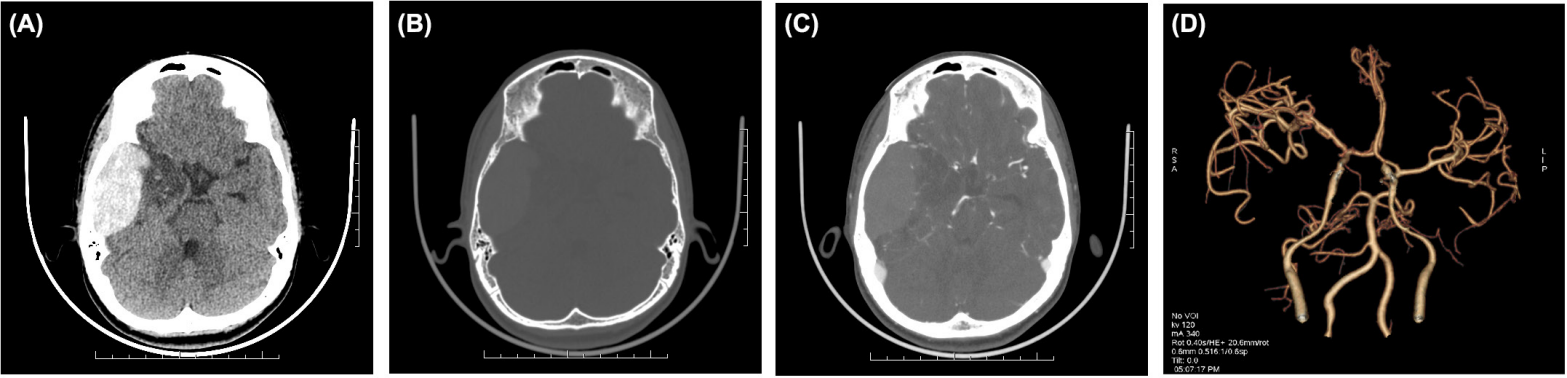
(A) Non‐contrast computed tomography (NCCT) scan axial section of head, brain window demonstrates hyperdense blood attenuation extradural collection in the right temporal region with mass effect and effacement of ipsilateral lateral ventricle and sulcal spaces. (B) Non‐contrast computed tomography (NCCT) scan axial section of head, bone windows do not show any fracture or bone pathologies. (C) Contrast enhanced computed tomography (CECT) arterial phase demonstrate displacement of cranial vessels due to extradural collection. No any vascular abnormalities are noted. (D) Reconstructed Volume rendering technique (VRT) shows no any abnormality.

A 16‐year‐old male patient previously diagnosed with Sickle Cell Beta Thalassemia who was on hydroxyurea presented with history of severe headache and vomiting for 3 days. Although there was past history of vaso‐occlusive crisis in the patient, history of antecedent trauma was not given. On physical examination, patient was conscious and oriented with no any localizing signs. An emergency computed tomography (CT) scan of the head was performed which demonstrated a hyperdense blood attenuation extradural collection in the right temporal region with mass effect and effacement of ipsilateral lateral ventricle and sulcal spaces (Figure 1A). No evidence of any fracture or bony abnormalities were noted (Figure 1B). Cerebral angiography did not show any vascular abnormalities (Figure 1C,D). Blood examination demonstrated hemoglobin 7.9 gm/dL, total bilirubin of 4.65 mg/dL, and direct bilirubin of 0.88 mg/dL. Platelet count, PT, aPTT, and INR were within normal limit. A craniotomy was performed with evacuation of blood. Patient recovered completely and had no complication on follow‐up.

## 3 DISCUSSION

Sickle cell disease (SCD) and its variants encompass a group of autosomal recessive disorders resulting from a single nucleotide polymorphism in the beta globin gene. Although considered rare in Nepal, recent studies have demonstrated increased prevalence of SCD as well as SCD along with other genetic hemoglobinopathies such as beta Thalassemia.^4^ Although there has been a tremendous advance in the understanding of inherited disorders of hemoglobin at molecular level, they are still under recognized resulting in significant morbidity as well as mortality.^1^

Central nervous system involvement is the most dreaded manifestation of SCD with significant morbidity and mortality. The various manifestations include chronic headache, epilepsy, ischemic or hemorrhagic stroke, cognitive impairment due to chronic anemia, Moyamoya disease, hypoxia, and silent infarcts.^2^ Although stroke is commonly encountered in the African and African American population, in our Nepalese population stroke is rarely documented.^4^, ^5^, ^6^

Spontaneous extradural hematoma is a rare complication of SCD with few cases reported in the literature.^7^, ^8^ Majority of the cases are from the African and Middle Eastern population, with no any case reported in the English literature affecting the Tharu population of Nepal. Mortality in a case series of spontaneous extradural hematoma has been reported to be almost 20%.^9^ Although calvarial infarction is a common occurrence in SCD, exact pathogenesis leading to spontaneous EDH is less understood, various mechanism are suggested as bone ischemia and infarction leading to extracranial or dural periosteal elevation, disruption of the bone margin and vessel wall resulting into bleeding into the epidural space; ischemia and spontaneous rupture of epidural vessels adjacent to infarcted bone; insufficient venous drainage resulting in venous congestion, rupture of veins, as well as edema and hemorrhage. Chronic medullary hematopoiesis resulting into proliferation of hematopoietic skull tissue with disruption of the skull margins, leading to blood and tissue extravasation into the epidural space has been implicated in majority of cases.^3^ In our case, no any underlying conditions and any calvarial bony changes were noted. Similar to our case, Shah et al also report a case where the cause of extradural hematoma could not be elucidated.^10^

## 4 CONCLUSION

Although neurological complications are rare in SCD patients in our region, it is instrumental in investigating individuals presenting with severe headache for a possible neurosurgical emergency. Furthermore, a possibility of SCD needs to be ruled out in targeted population presenting with neurosurgical emergencies.

## AUTHOR CONTRIBUTIONS

All authors have participated in writing and reviewing the manuscript.

## CONFLICT OF INTEREST STATEMENT

The authors declare that they have no competing interests.

## CONSENT

Written informed consent was obtained from the patient to publish this report in accordance with the journal's patient consent policy.

## Data Availability

The data that support the findings of this study are available on request from the corresponding author. The data are not publicly available due to privacy or ethical restrictions.

## References

[1] Weatherall DJ , Clegg JB . Inherited haemoglobin disorders: an increasing global health problem. Bull World Health Organ. 2001;79(8):704‐712. 11545326 PMC2566499

[2] Mengnjo MK , Kamtchum‐Tatuene J , Nicastro N , Noubiap JJ . Neurological complications of sickle cell disease in Africa: protocol for a systematic review. BMJ Open. 2016;6(10):e012981. 10.1136/bmjopen-2016-012981 PMC5073509 27798028

[3] N'Dri Oka D , Tokpa A , Bah A , Derou L . Spontaneous intracranial extradural hematoma in sickle cell disease. J Neurol Surg Rep. 2015;76(1):e97‐e99. 26251822 10.1055/s-0035-1544953 PMC4520986

[4] Pande R , Ghimire PG , Chand PB . Sickle cell disease in Western Nepal. Nepal J Med Sci. 2019;4(1):15‐19.

[5] Patra SK , Mishra SS , Das S . A rare case of spontaneous bilateral extradural hematoma in a sickle cell disease child. J Pediatr Neurosci. 2012;7(1):77‐78. 22837791 10.4103/1817-1745.97636 PMC3401667

[6] Jacob M , Saunders DE , Sangeda RZ , et al. Cerebral infarcts and vasculopathy in Tanzanian children with sickle cell anemia. Pediatr Neurol. 2019;107:64‐70. 32111561 10.1016/j.pediatrneurol.2019.12.008

[7] Hettige S , Sofela A , Bassi S , Chandler C . A review of spontaneous intracranial extradural hematoma in sickle‐cell disease. Acta Neurochir (Wien). 2015;157(11):2025‐2029. discussion 9. 26374442 10.1007/s00701-015-2582-6

[8] Azhar MJ . Extradural hemorrhage: a rare complication and manifestation of stroke in sickle cell disease. Oman Med J. 2010;25(4):e017. 28845220 10.5001/omj.2010.97 PMC5556317

[9] Hamm J , Rathore N , Lee P , et al. Cranial epidural hematomas: a case series and literature review of this rare complication associated with sickle cell disease. Pediatr Blood Cancer. 2017;64(3):e26237. 10.1002/pbc.26237 27618802

[10] Shah D , Reddy H , Kumar S , Acharya S . Sickle cell disease presenting as extradural hematoma: an extremely rare fatal crisis. Cureus. 2022;14(7):e27004. 36000137 10.7759/cureus.27004 PMC9390951

